# The Story of the Insane from Year to Year

**Published:** 1899-11-25

**Authors:** 


### THE STORY OF THE INSANE FROM YEAR TO YEAR.

Twelfth Series.

### THE THREE COUNTIES ASYLUM AT ARLSEY.

At the beginning of the year 1898 there were in residence 1,085 patients, and at the end of December the number had risen to 1,105. There were 220 first admissions, 35 re-admissions, 114 recoveries, 11 discharged relieved, and 118 deaths. Calculated in the usual way in asylums, the recoveries stand at 39·1 per cent., and the deaths at 10·8 per cent. As regards the recoveries this percentage is a very fair one, and as regards the deaths it is just a little above the average. Of the deaths during the year 32 were caused by cerebral and spinal diseases, 4 by pneumonia, 1 by bronchitis, and 22 by consumption. Various moral causes account for the insanity in 53 of the admissions, intemperance in drink for 25, previous attacks for 41, and hereditary influence for 98. It will be seen that heredity and drink accounts for more than 50 per cent. of the insanity. Both of these are preventable causes, and yet we cannot learn that anything is being done to put a stop to the marriage of people with an insane taint—and we fear not much is being done to stem the tide of drunkenness. We are pleased to be able to congratulate attendants John Newberry and George Berridge on obtaining their pensions—the former £39 and the latter £41 12s. It cannot be said that either of these is very much to live on, but if single men they may have saved money during their period of service. It is much to be regretted that attendants and nurses do not more frequently join the National Pension Fund. The committee say, "the management of the asylum has been carried on by Dr. Swain to the full satisfaction of the Visiting Committee." The weekly cost of maintenance is 8s. 3⅝d. per head.

### SUFFOLK COUNTY ASYLUM AT MELTON.

On January 1st, 1898, the asylum contained 612 patients, and on January 31st the number had fallen to 605. There were 141 first admissions, 28 readmissions, 63 recoveries, 10 discharged relieved, and 64 deaths. The recoveries, calculated on the admissions, stand at 40·91 per cent., and the deaths, calculated on the average number resident, stand at 10·32. Both of these percentages are above the average in asylums. Cerebral and spinal diseases account for 12 of the deaths, bronchitis for 2, pneumonia for 2, and consumption for 13. Of the year's admissions 37 were driven insane by such moral causes as domestic trouble, religion, and adverse circumstances; 30 by intemperance in drink; and 51 by hereditary tendency. Strictly speaking, the asylum has only space for 591 patients, but, as will be seen from the figures quoted above, the number is in excess of this. To remedy this overcrowding and provide for future contingencies it has been decided to build additional blocks for 280 patients. The asylum is a very old one, and it has also been decided to make considerable alterations, which will bring the building up to date. There has been a great improvement in the sanitary condition of the buildings, and the committee say this is principally due to the great attention bestowed upon it by the medical superintendent, Dr. Whitwell. Another assistant medical officer has been appointed, so we may now hope for original researches on the part of the medical staff. The average weekly cost of maintenance is 9s. 2⅝d. per head.

